# Profiles and diagnostic value of intestinal microbiota in schizophrenia patients with metabolic syndrome

**DOI:** 10.3389/fendo.2023.1190954

**Published:** 2023-07-27

**Authors:** Mengjuan Xing, Hui Gao, Lili Yao, Li Wang, Chengfang Zhang, Liping Zhu, Donghong Cui

**Affiliations:** ^1^ Department of General Psychiatry, Shanghai Mental Health Center, Shanghai Jiao Tong University, School of Medicine, Shanghai, China; ^2^ The First Minzheng Mental Health Center, Shanghai, China; ^3^ Shanghai Pudong New Area Mental Health Center, Tongji University School of Medicine, Shanghai, China; ^4^ Shanghai Key Laboratory of Psychotic Disorders, Shanghai Mental Health Center, Shanghai Jiao Tong University, School of Medicine, Shanghai, China; ^5^ Brain Science and Technology Research Center, Shanghai Jiao Tong University, Shanghai, China

**Keywords:** metabolic syndrome, intestinal microbiota, 16S rRNA, schizophrenia, type 2 diabetes, dyslipidemia

## Abstract

**Aims/hypothesis:**

It is widely thought that the intestinal microbiota plays a significant role in the pathogenesis of metabolic disorders. However, the gut microbiota composition and characteristics of schizophrenia patients with metabolic syndrome (MetS) have been largely understudied. Herein, we investigated the association between the metabolic status of mainland Chinese schizophrenia patients with MetS and the intestinal microbiome.

**Methods:**

Fecal microbiota communities from 115 male schizophrenia patients (57 with MetS and 58 without MetS) were assessed by 16S ribosomal RNA gene sequencing. We assessed the variations of gut microbiome between both groups and explored potential associations between intestinal microbiota and parameters of MetS. In addition, the Phylogenetic Investigation of Communities by Reconstruction of Unobserved States (PICRUSt) based on the KEGG database was used to predict the function of intestinal microbiota. We also conducted Decision Tree Analysis to develop a diagnostic model for the MetS in patients with schizophrenia based on the composition of intestinal microbiota.

**Results:**

The fecal microbial diversity significantly differed between groups with or without MetS (α-diversity (Shannon index and Simpson index): p=0.0155, p=0.0089; β-diversity: p=0.001). Moreover, the microbial composition was significantly different between the two groups, involving five phyla and 38 genera (p<0.05). In addition, a significant correlation was observed between the metabolic-related parameters and abundance of altered microbiota including HDL-c (r2 = 0.203, p=0.0005), GLU (r2 = 0.286, p=0.0005) and WC (r2 = 0.061, p=0.037). Furthermore, KEGG pathway analysis showed that 16 signaling pathways were significantly enriched between the two groups (p<0.05). Importantly, our diagnostic model based on five microorganisms established by decision tree analysis could effectively distinguish between patients with and without MetS (AUC = 0.94).

**Conclusions/interpretation:**

Our study established the compositional and functional characteristics of intestinal microbiota in schizophrenia patients with MetS. These new findings provide novel insights into a better understanding of this disease and provide the theoretical basis for implementing new interventional therapies in clinical practice.

## 1 Introduction

Schizophrenia patients have been reported to have a lifespan of 10-15 years shorter than the general population (1). Cardiovascular disease (CVD) is the leading cause of premature mortality in patients with schizophrenia (2). Over the years, metabolic syndrome (MetS) has become a public health concern and contributes to adverse effects and poor CVD outcomes among schizophrenia patients (3). It is widely acknowledged that MetS represents the clustering of several conditions, including abdominal obesity, hypertension, hyperglycemia, and hyperlipidemia. Varying incidence of MetS has been reported in the literature ranging from 10.1% to 69.3% in patients with schizophrenia (4, 5). Current evidence suggests MetS can contribute to cognitive impairment and dementia (6). Accordingly, it poses a severe public health challenge worldwide (4). An increasing body of evidence suggests that lifestyle habits (7), physical activity, genetic predispositions (8), immune abnormalities (9), and antipsychotic medicine (10) are associated with the pathogenesis of MetS in schizophrenia patients.

Over the last few years, several studies have reported that the microbiota of the human intestine have played a vital role in the pathogenesis of MetS (11). The ratio between human cells and bacteria is estimated at 1:1, whereas the genome of the bacterial strain is 100 times greater than in humans (12). It has been proposed that the microbiota have been an “essential organ” of the human body and play a vital role in human health and disease (13). Dysbiosis of the intestinal flora causes metabolic problems by affecting the energy balance of the host, eating behavior, and chronic inflammation (14). A previous study reported that gut microbiota promoted the absorption of monosaccharides in the host’s intestinal lumen and induced new hepatic lipogenesis, leading to increased body fat content and insulin resistance (15). Kalliomäki et al. found a decrease in Bifidobacteria and an increase in Staphylococcus aureus in the intestine of overweight/obese children during the first year of life compared to normal-weight children after a 7-year prospective study and concluded that abnormalities in the intestinal flora might occur before the development of obesity (16). Other studies showed that obesity was associated with a proportional change of Firmicutes/Bacteroidetes (F/B) and level of alternate Proteobacteria in the gastrointestinal tract^14^. Although overwhelming evidence substantiates that the intestinal microbiota are fundamentally related to human health and disease, several inconsistencies have been reported (17). Few studies have focused on the role and characteristics of the intestinal microbiota in schizophrenia patients suffering from MetS. In contrast, there are significant differences in the distribution of intestinal flora in different metabolic states. Accordingly, it is essential to investigate the characteristics of gut flora in schizophrenia patients with MetS.

**Figure 1:**
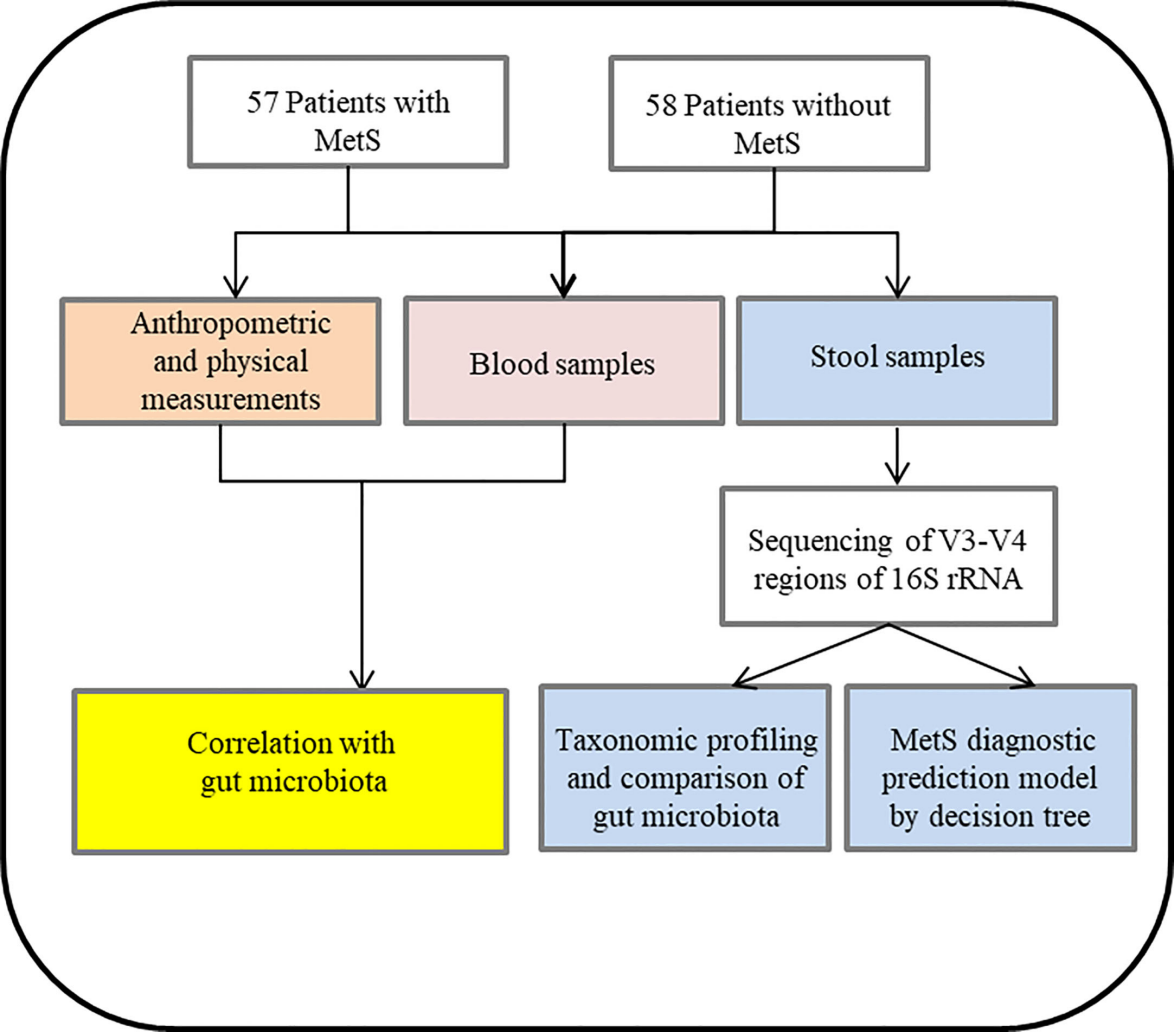
Flow chart and enrolled subjects in the current study.

This study aimed to characterize the gut microbiota of schizophrenia patients suffering from MetS. In addition, we examined the possible association between the gut microbiota and the clinical parameters of MetS. Moreover, we developed a model of intestinal microbiota to predict MetS, proposing a novel treatment strategy for schizophrenia patients with MetS. The flow chart see **Figure 1**.

## 2 Methods

### 2.1 Sample collection

#### 2.1.1 Study subjects

The research protocol was approved by the Shanghai First Minzheng Mental Health Center (study number: YJZXLL2022022), and all participants were recruited between June 2021 and December 2021. Informed written consent was provided by all patients and/or their guardians. A total of 115 subjects (without MetS group, n=58; with MetS group, n=57) were recruited for this current study.

All patients were recruited in this study based on the following criteria: 1) all patients met the diagnosis of schizophrenia according to the Diagnostic and Statistical Manual of Mental Disorders, fifth edition (DSM-5); 2) patients were continuously hospitalized for at least ten years; 3) patients who received antipsychotic monotherapy treatment. 4) Patients were older than 55 years old. Patients were excluded for the following reasons: 1) Severe physical illness or infection; 2) Diarrhea within two months before recruitment; 3) Use of probiotics or antibiotics in the three months before recruitment; 4) Co-morbidities or other psychiatric disorders that meet the DSM-V diagnostic criteria.

#### 2.1.2 Clinical data collection

We collected socio-demographic and clinical information through structured interviews and medical records. The primary medical records include height, weight, body mass index (BMI), waist circumference (WC), and resting blood pressure. Biochemical indicators such as fasting blood glucose (FBS) and lipid profile were performed from venous blood following 8 hours of fasting. The Positive and Negative Syndrome Scale (PANSW) was used to evaluate the patient’s psychiatric symptoms. All researchers involved in this study received training in structuring clinical information collection.

#### 2.1.3 Definition of MetS

The definition of MetS was consistent with our previous study (18). A diagnosis of metabolic syndrome was established when 3 or more of the following criteria were met: 1) WC≥90cm; 2) TG level ≥150mg/dL; 3) HDL-c level ≤40mg/dL; 4) DBP ≥85mmHg and/or SBP ≥130mmHg; 5) FBS levels ≥100mg/dL^32^.

#### 2.1.4 DNA extraction, PCR amplification, and sequencing

Fresh fecal samples were collected from each study subject with sterile collection containers and stored at -80°C. The CTAB (hexadecyltrimethylammonium bromide) method was used to extract the total genome DNA of fecal samples. We monitored the DNA concentration and purity on 1% agarose gel, and then DNA was diluted to 1ng/µL using sterile water.

The V3-V4 region of 16S ribosomal RNA genes was amplified using a specific primer 341F (5’-CCTAYGGGRBGCASCAG-3’) and 806R (5’-GGACTACNNGGGTATCTAAT-3’) with the barcode. All PCR reactions were carried out with 15 µL of Phusion^®^ High-Fidelity PCR Master Mix (New England Biolabs), two µM of forward and reverse primers, and about ten ng templates DNA. Thermal cycling consisted of an initial denaturation at 98°C for 1 min, followed by 30 cycles of denaturation at 98°C for 10 s, annealing at 50°C for 30 s, and elongation at 72°C for 30 s, with a final extension at 72°C for 5 min.

The same volume of 1XTAE buffer was mixed with PCR products and underwent electrophoresis on 2% agarose gel for detection. PCR products were mixed in equidensity ratios. Then, the mixture of PCR products was purified with Qiagen Gel Extraction Kit (Qiagen, Germany).

Sequencing libraries were generated usingTruSeq^®^ DNA PCR-Free Sample Preparation Kit (Illumina, USA) following the manufacturer’s recommendations, and index codes were added. The library quality was assessed on the Qubit@ 2.0 Fluorometer (Thermo Scientific). At last, the library was sequenced on an Illumina NovaSeq platform, and 250 bp paired-end reads were generated.

#### 2.1.5 Bioinformatics analysis

The analysis was performed according to the “Atacama soil microbiome tutorial” of Qiime2docs and customized program scripts (https://docs.qiime2.org/2019.1/). Briefly, raw data FASTQ files were imported into the QIIME2 systems, and a series of operations were performed to obtain the feature table of amplified sequence variant (ASV) (19). The QIIME2 feature-classifier plugin was then used to align ASV sequences to a pre-trained GREEN GENES 13_8 99% database (trimmed to the V3V4 region bound by the 338F/806R primer pair) to generate the taxonomy table (20). Any contaminating mitochondrial and chloroplast sequences were filtered using the QIIME2 feature-table plugin. Methods such as ANCOM, ANOVA, Kruskal Wallis, LEfSe, and DEseq2 were employed to identify the bacteria with different abundance among samples and groups (21, 22). Bacterial diversity was determined by alpha diversity (observed OTUs, Chao1 richness estimator, Shannon diversity index, and Faith’s phylogenetic diversity index) and Beta diversity (principal coordinate analysis (PCoA) (23). Analysis of variance (ANOVA) was used to evaluate the α-diversity among the different groups, and PERMANOVA testing was performed for microbial community clustering (PCoA) by Bray Curtis, unweighted UniFrac, and weighted UniFrac. Redundancy analysis (RDA) was performed to reveal the association of microbial communities concerning metabolic parameters based on the relative abundance of microbial species from different taxa levels using the R package “vegan.” In addition, the potential KEGG Ortholog (KO) functional profiles of microbial communities were predicted with PICRUSt (24). Unless specified above, parameters used in the analysis were set as default.

#### 2.1.6 Statistical analyses

IBM SPSS (version 22.0) was used to manage and analyze the demographic and clinical data. We tested the homogeneity of variances using Levene’s test. Comparisons between groups were performed with variance (ANOVA) or Mann-Whitney U tests for quantitative variables. We tested the differences between the two groups and the operational taxonomic units (OTUs), phylum, and genus levels. The relative abundance of each taxon in the MetS group versus the without MetS group, the Observed_otus, Shannon index, and Simpson index were calculated using the Analysis of Composition of Microbiomes (ANCOM) and Kruskal-Wallis rank sum test in R software. STAMP software was used to assess the gut microbiota composition of phylum and genus level using default parameters, and the significance was set at p<0.05. Correlations between metabolic parameters and genera were calculated using Spearman’s rank-correlation analysis based on the assumption that there was a non-linear relationship between the examined variables. We performed a linear discriminant effect size (LEfSe) analysis to identify the differentially abundant taxa between the two groups. At first, features with significant differential abundance were identified using linear discriminant analysis (LDA), and then the effect size of each feature was calculated. The functional differences associated with predicted KEGG functional pathways between the MetS group and non-MetS group were assessed by an one-way ANOVA followed by Tukey-Kramer multiple comparisons. A diagnostic prediction model for MetS was constructed using receiver operating characteristic curves (ROC) and Kolmogorov-Smirnov curve (KS). The area under ROC (AUC), as well as the value of true positive rate (TPR) and false positive rate (FPR), were calculated to assess the diagnostic performance of the model with python. A p-value <0.05 was statistically significant.

## 3 Results

### 3.1 Clinical characteristics of schizophrenia patients with and without MetS

We recruited a total of 115 male schizophrenia patients that were divided into MetS (n=57) and non-MetS (n=58) groups, and their clinical characteristics were compared, including age, metabolic-related parameters such as WC, BMI, BP, GLU and lipid profile, and PANSS scores (**Table 1**). The two groups did not significantly differ in age, WC, DBP, TC, and PANSS scores. In contrast, the MetS group displayed significantly higher BMI, GLU, SBP, TG, and LDL-c; and a lower level of HDL-c than the non-MetS group (p_s_<0.05).

**Table 1:** Demographic and clinical parameters of schizophrenia patients with and without MetS.

	With MetS (n=57)	Without MetS (n=58)	*p*
Age (ages)	66.09 ± 7.74	67.81 ± 8.23	0.25
WC (cm)	96.56 ± 8.98	83.03 ± 9.34	0.538
BMI (kg/m^2^)	25.47 ± 3.11	20.96 ± 3.17	<0.001**
GLU (mmol/L)	5.36 ± 0.89	4.77 ± 0.52	<0.001**
SBP (mmHg)	132.19 ± 12.47	124.28 ± 14.07	0.002**
DBP (mmHg)	75.96 ± 8.35	72.95 ± 8.43	0.056
HDL-c (mmol/L)	0.89 ± 0.19	1.13 ± 0.22	<0.001**
TG (mmo/L)	1.60 ± 0.84	0.96 ± 0.50	<0.001**
TC (mmol/L)	4.61 ± 0.75	4.53 ± 0.86	0.295
LDL-c (mmol/L)	2.83 ± 0.54	2.48 ± 0.65	0.02*
PANSS			
T scores	71.19 ± 14.64	73.96 ± 15.90	0.333
P scores	10.44 ± 4.78	10.93 ± 4.76	0.581
N scores	25.58 ± 6.53	27.76 ± 6.05	0.066
G scores	35.167.89	36.03 ± 6.74	0.523

### 3.2 Altered gut microbial diversity between the MetS group and non-MetS group

**Figure 2:**
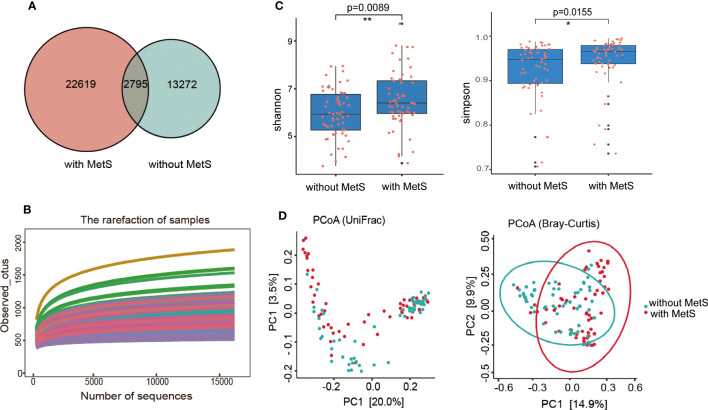
The α-diversity and β-diversity indices of the fecal microbiome in schizophrenia patients with MetS and without Mets. **(A)** Venn plot of OUT, **(B)** Dilution curve of the α-diversity index. **(C)** The Shannon and Simpson indexes are based on OUT counts between the two groups (p=0.0155, p=0.0089). OUT, operational taxonomic units. **(D)** PCoA plots of bacterial β-diversity based on the weighted UniFrac distance (left panel) and Bray-Curtis dissimilarity (right panel) were analyzed between the two groups (both p=0.001) *: p <0.05, **: p<0.001.

We conducted high-throughput sequencing of the V3-V4 regions of 16S rRNA genes and obtained the number of operable taxonomic units (OTUs). Then, we characterized the bacterial gut microbiota of all samples. The number of overlapping OTUs (n=2795) between the two groups was visualized in a Venn plot. 22619 OTUs were uniquely found in the MetS group, and 13272 OTUs in the non-MetS group (**Figure 2A**). During rarefaction analysis, the estimated OTU richness of all samples was mainly close to saturation, indicating that the OTU richness of all models was sufficient for the subsequent analysis (**Figure 2B**).

We used well-established indexes such as Shannon and Simpson indexes to estimate the gut microbial α-diversity between the two groups, which showed that the MetS group exhibited significantly increased bacterial gut microbial diversity than the non-MetS group (p=0.0089 for the Shannon index, and p=0.0155 for the Simpson index; by Wilcoxon rank-sum Test, **Figure 2C**). The weighted Unifrac distances and Bray-Curtis dissimilarity were used to calculate the β-diversity in the gut microbiota of schizophrenia patients between the two groups. Principal coordinates analysis (PCoA) found that the gut microbiota were significantly different between the two groups (both P=0.001, by permutational MANOVA (PERMANOVA)) (**Figure 2D**). These findings indicated that the gut microbiota diversity was significantly different between the two groups.

### 3.3 Differences in the gut microbiome composition between the MetS group and non-MetS group

**Figure 3:**
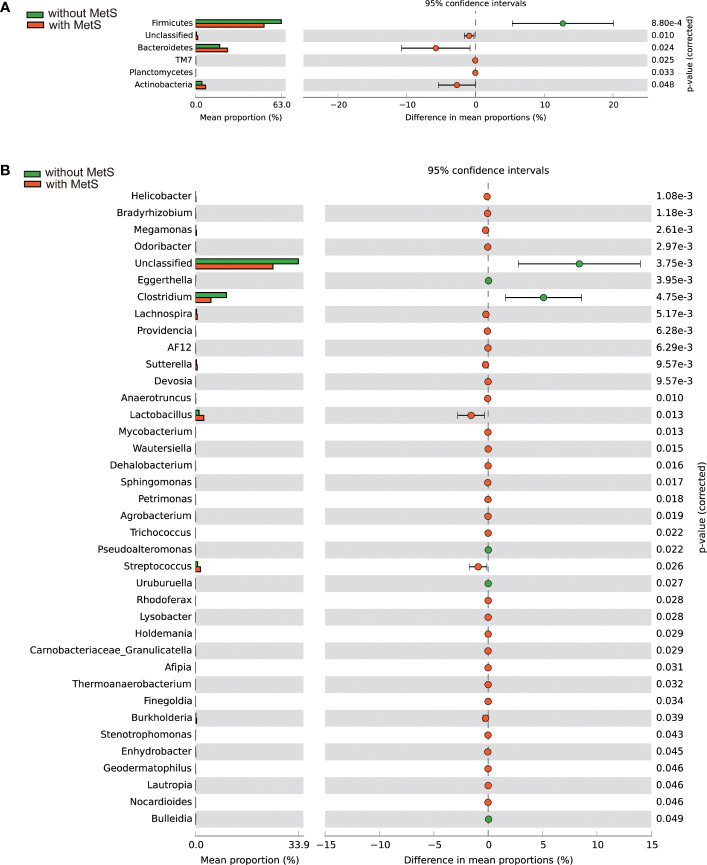
Gut microbiota composition alterations at the phylum and genus levels between the Mets and non-MetS groups. **(A)** The relative abundance of 5 phyla was significantly different between the two groups. **(B)** The relative abundance of 38 genera was significantly different between the two groups.

Phylotype abundance in both groups at the phylum level and genus level were compared. We analyzed the fecal bacterial microbiome abundance using the Wilcoxon rank-sum test with the Benjamini-Hochberg method (**Figure 3**). At the phylum level (**Figure 3A**), there were five significant microbiota differences between the two groups, including Firmicutes, Bacteroidetes, TM7, Planctomycetes, and Actinobacteria. In addition, the abundance of Firmicutes was substantially higher in the non-MetS group (p=8.80e-4). In comparison, the abundance of the other four phyla was significantly higher in the MetS group (p_s_ <0.05). At the genus level, there were 37 genera with significant differences between the two groups (**Figure 3B**). The abundance of five genera such as Clostridium, Eggerthella, Pseudoalteromonas, Uruburuella, and Bulleidia was significantly higher in the non-Mets groups (p_s_ <0.05), and the abundance of the other 32 genera was significantly higher in the MetS group (p_s_ <0.05), suggesting that the composition of gut microbiota was altered significantly between the two groups.

### 3.4 Taxonomic alterations in gut microbiota between the MetS group and non-MetS group

To explore the presence and effect size of region-specific OTUs between the MetS group and without the MetS group, we used the linear discriminant analysis (LDA) effect size (LEfSe) analysis to supervise the difference comparison of gut microbiota between the two groups. We applied LDA LEfSe analysis to identify critical taxonomic differences and gut microbiota between the two groups, with a log LDA score threshold set as 3.0. A total of 45 taxa (from phylum to species) significantly differed between the two groups.

**Figure 4:**
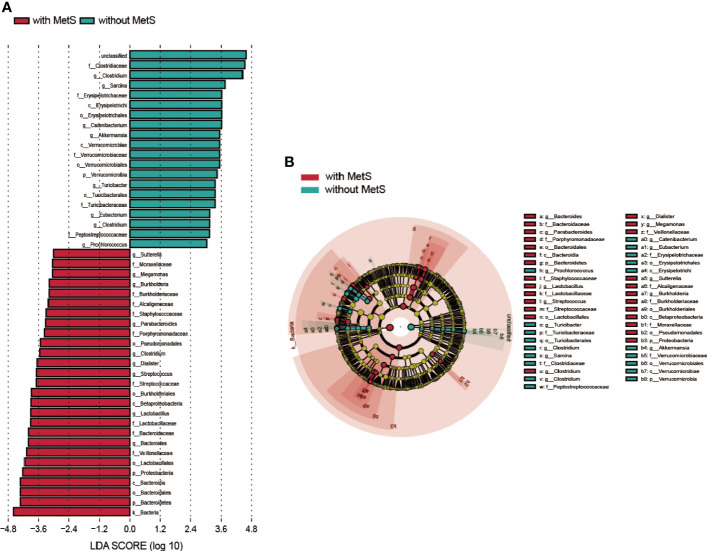
Taxonomic differences of fecal microbiota in schizophrenia patients with MetS and without MetS. **(A)** A Linear discriminant analysis (LDA) effect size (LEfSe) analysis revealed significant bacterial differences in fecal microbiota between the two groups. LDA scores (log 10) >3 and p<0.05 are listed. **(B)** Cladogram using the LEfSe method indicating the phylogenetic distribution of fecal microbiota associated with the MetS and non-MetS groups.

We found the relative abundances of the Bacteroides genus, Lactobacillus genus, Lactobacillus Streptococcus genus, Dialister genus, Clostridium genus, Parabacteroides genus, Burkholderia genus, Megamonas genus, and Sutterella genus were higher in the MetS group. In contrast, the relative abundances of Clostridium genus, Sarcina genus, Catenibacterium genus, Akkermansi genus, Turicibacter genus, Eubacterium genus, Clostridium genus, and Prochlorococcus genus were higher in the non-MetS group (**Figure 4**).

### 3.5 Fecal microbiota alteration is associated with metabolic parameters

**Figure 5:**
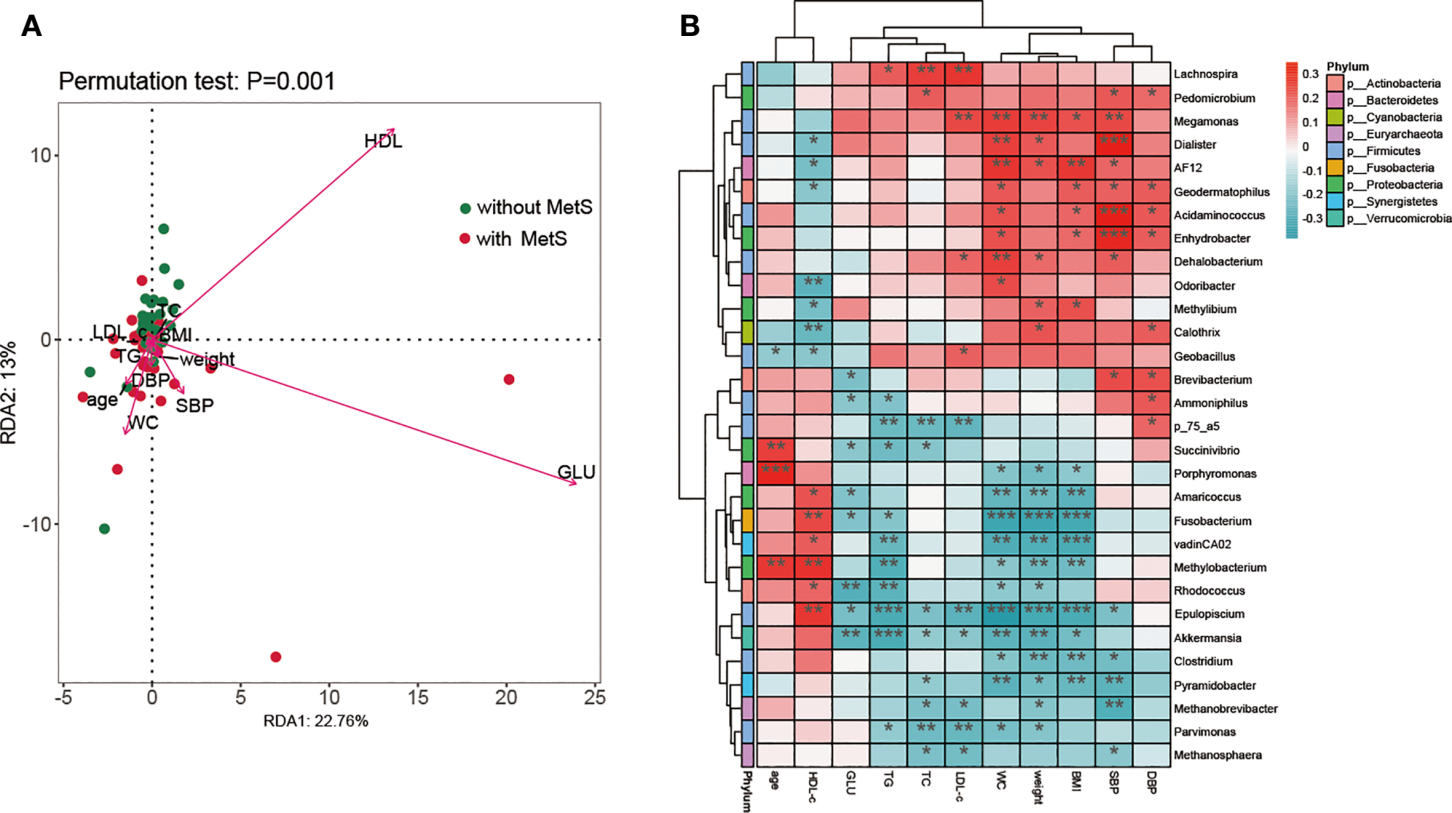
The association analysis of fecal microbiota composition and metabolic parameters. **(A)** A biplot of redundancy analysis (RDA axis one versus axis 2) of fecal microbiota data, constrained by related metabolic factors including WC, weight, GLU, HDL, HDL-c, TG, BMI, SBP, DBP, TC, and LDL-c. **(B)** Heatmaps of correlations between differentially abundant microbiota genera and metabolic-related factors. *: p <0.05, **: p<0.001, ***:p< 0.0001.

It has been established that environmental changes generally influence the functionality and pathophysiology of fecal microbiota. Thus, we applied RDA analysis to evaluate the relationship between gut microbiota and metabolic-related parameters in this current study. The amount of explanatory variation by all the metabolic-related variables was 65.76% which suggested that the metabolic-related factors could significantly alter the population of the fecal microbiome at the genus level. According to the Monte Carlo permutation test, the metabolic-related parameters HDL-c (r2 = 0.203, p=0.0005), GLU (r2 = 0.286, p=0.0005), and WC (r2 = 0.061, p=0.037) were significantly associated with the distribution of bacterial taxa in the constrained ordination model (**Figure 5A**). We used Spearman’s correlation analysis to explore the relationship between the abundances of thirty significantly altered gut microbial genera and changes in metabolic-related indices among all the participants. We found close associations between genera and glucose metabolism, lipid profiles, BMI, BP, and other metabolic-related parameters. TC and LDL-c showed a strong positive association with the Lachnospira genus and a negative correlation with the Parvimonas genus. TG was negatively correlated with the Epulopiscium and Akkermansia genera. In contrast, HDL-c was positively correlated with Fusobacterium, Methylobacterium, and pulopiscium genera and negatively associated with Odoribacter and Calothrix genera. Moreover, serum glucose was negatively associated with Rhodococcus and Akkermansia genera. SBP showed a strong positive correlation with Dialister, Acidaminococcus, and Enhydrobacter genera and a negative association with Pyramidobacter and Methanobrevivacter genera. Finally, BMI, WC, and weight displayed a similar relationship with these thirty genera; they were positively associated with Megamonas and AF12 genera and negatively associated with Jusobacterium and Epulopiscium genera (**Figure 5B**). Overall, our findings suggest that the genera of Dialister, Acidaminococcus, Enhydrobacter, Fusobacterium, Epulopiscium, Epulopiscium, Akkermansia, Rhodococcus, and Methylobacterium are closely associated with the development of MetS.

### 3.6 Functional prediction of gut microbiota

**Figure 6:**
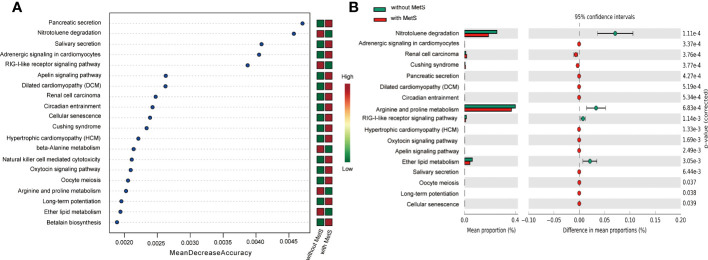
KEGG pathway analysis of Mets and non-Mets groups. **(A)** Enrichment analysis of KEGG signaling pathways. **(B)** Variance analysis of KEGG between the two groups.

First, we applied PICRUSt2 to predict the KEGG signaling pathway of the amplicon genes, followed by a random forest approach to enrich the amplicon genes and identify the top 20 functionally significant signaling pathways (**Figure 6A**). Furthermore, intergroup variation analysis on these 20 signaling pathways yielded 16 significant signaling pathways (**Figure 6B**).

### 3.7 MetS diagnostic prediction model based on taxonomic compositions of the gut microbiota

**Figure 7:**
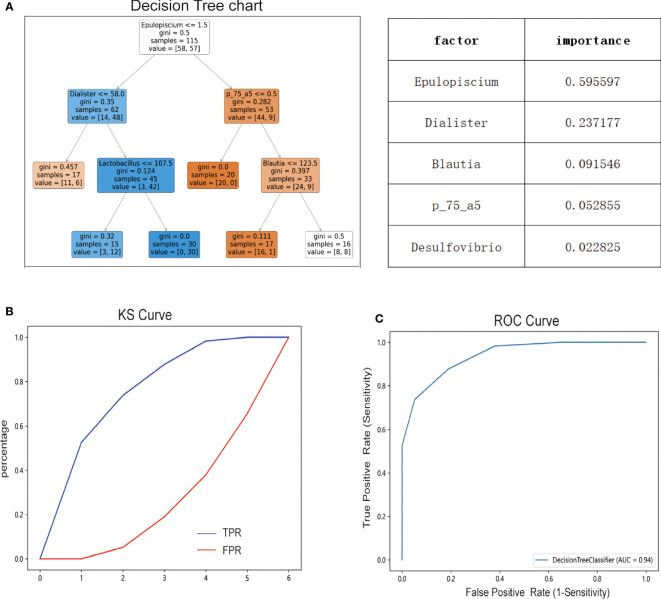
Diagnostic prediction model for MetS based on taxonomic compositions of the gut microbiota. **(A)** Predictive modeling of metabolic syndrome diagnosis using decision trees. **(B)** Efficiency of decision tree model evaluated by Ks curve. **(C)** Efficiency of decision tree model evaluated by ROC curve.

The decision tree is a differential classification model using Gini coefficients to classify two groups of cases. As shown in **Figure 7A**, the decision tree model had a depth of four with a total of eleven nodes, including six terminal nodes. The model showed that variables that significantly contributed to the diagnosis were Epulopiscium, Dialister, Blautia, p_75_a5, and Desulfovibrio genus. The model Epulopiscium ≤ 1.5 + Dialister ≤ 58.0 yielded a predicted prevalence probability of 6/17 (overall sample prevalence is 57/115), which was lower than observed in our samples. Likewise, the prevalence probability was 12/15 for Epulopiscium ≤1.5 + Dialister > 58.0 + Lactobacillus ≤107.5. At Epulopiscium ≤ 1.5 + Dialister>58.0 + Lactobacillus >107.5, the prevalence probability was 30/30. Both models yielded a much greater prevalence than observed in our samples. The models Epulopiscium>1.5 + p_75_a75 ≤ 0.5, Epulopiscium>1.5 + p_75_a75>0.5 + Blautia ≤ 123.5, or Epulopiscium>1.5 + p_75_a75>0.5 + Blautia>123.5 yielded a prevalence of 0/20, 1/17, and 8/16 respectively, lower than observed in our samples. KS curve analysis was used to evaluate the efficiency of the decision tree model by describing cumulatively diseased and non-diseased samples. Indeed, the more obvious the distinction between the two curves, the stronger the model’s prediction accuracy. The KS curve (**Figure 7B**) showed that the two groups of samples were clearly separated. ROC curve analysis was also applied to evaluate the prediction efficiency of the decision tree model. The area under the ROC curve of the model in **Figure 7C** was 0.94, indicating that the model yielded a good performance and could better distinguish MetS patients from non-Mets patients.

## 4 Discussion

Growing evidence suggests that alterations in the gut microbiome are associated with metabolic disorders, inflammatory, neurodegenerative diseases, and cancer among human beings (13, 25). There is a rich literature available suggesting that the fecal microbiome plays a vital role in the pathogenesis of MetS among the general population (26). To our knowledge, few studies have hitherto characterized the relationship between gut microbiota and schizophrenia patients with metabolic syndrome. Herein, we depicted the community structure of intestinal microbiota in schizophrenia patients with MetS utilizing 16S rRNA gene sequencing. Importantly, we found intestinal microbiota composition alterations in male schizophrenia individuals with metabolic syndrome from the Chinese mainland population. In this respect, at the phylum level, the relative abundance of five gut microbiota differed significantly between the MetS and non-MetS groups, and at the genus level, the relative abundance of 38 intestinal microbiomes differed significantly between the two groups. Moreover, we found a significant correlation between the gut microbiota and the components of MetS and used PICRUSt2 to predict the differences in KEGG signaling pathways between the two groups and found 16 signaling pathways with significant differences. Based on the microbial signature, we used decision tree analysis to establish a diagnostic prediction model for MetS. Importantly, we found that Epulopiscium, Dialister, Blautia, P_75_a5, and Desuflvibrio genus were highly enriched in patients with schizophrenia and associated with the diagnosis of MetS. These findings provide clues to understanding this disease and identifying new targets or markers for diagnosis and intervention.

The fecal microbiome of schizophrenia patients with MetS exhibited significant alterations in richness and evenness, defined as the α-diversity index, compared with patients without MetS, consistent with a previous study that reported that the α-diversity of the gut microbiome increased in obese patients who underwent sleeve gastrectomy after three months^27^. In the present study, the β-diversity index exhibited significant differences in the microbiome structures between the MetS and non-MetS groups, consistent with previous studies suggesting the intestinal microbiota were altered in schizophrenia patients with MetS (27, 28). Likewise, microbial diversity alteration has been associated with other conditions, including Alzheimer’s disease, obesity, inflammatory bowel disease, and Parkinson’s disease (29, 30). Thus, a better understanding of intestinal microbiome variability between schizophrenia patients with and without MetS can potentially help assess and diagnose MetS early and provide timely treatment.

Current evidence suggests that the phylum Bacteroidetes and Firmicutes dominate healthy adults’ intestinal tract, representing more than 90% of the total community (31). This composition is unaffected by acute perturbations, as its plasticity allows a rapid return to its initial composition (32). Therefore, it is essential to characterize the bacterial communities involved in dysbiosis. Our study observed that Firmicutes and Bacteroidetes significantly differed between the two groups, and the abundance of Firmicutes decreased while Bacteroidetes considerably increased in the MetS group. Consistently, it has been shown that African children who lived in rural areas consuming a rich fiber diet showed higher proportions of Bacteroidetes and lowered Firmicutes (33). Moreover, some studies showed that adults with obesity and type 2 diabetes presented a higher abundance of Bacteroidetes. In contrast, Turnbaugh et al.’s study found that obese mice had a 50% decrease in Bacteroidetes and a proportional expander in Firmicutes and Archaea^34^. These findings suggest that intestinal microbiota composition is affected by many factors and that the outcome of these influences is subject to significant heterogeneity.

It has been established that gut microbiota have been a contributing factor to obesity (34). Interestingly, we found an alteration of the Bacteroidetes/Firmicutes ratio between the two groups, and the ratio declined in the MetS group. Over the years, much emphasis has been placed on the role of the Bacteroidetes/Firmicutes ratio (35), which is widely thought to have significant value for diagnosis and therapy in the future.

Most parameters related to MetS (such as BMI, HDL-c, TG, GLU, and SBP/DBP) were significantly different between the two groups. To explore the possible effect of the intestinal microbiome on schizophrenia patients and metabolic parameters, we performed correlation analyses between each genus’s abundance and metabolic parameters. HDL-c, GLU, and WC were significantly associated with the intestinal microbiome. HDL-c showed a significant positive correlation with Fusobacterium, Methylobacterium, and Epulopiscium and a significant negative correlation with Odoribacter and Calothrix. Akkermansia had a significant negative correlation with both GLU and WC. Consistently, previous animal studies illustrated that the gut microbiome influenced host TG levels and energy metabolism by increased lipoprotein lipase-mediated TG storage in adipose tissue (36, 37). However, they did not identify which microbiota influenced lipid and energy metabolism.

Karlsson et al. found that 66 fecal metagenomics gene clusters were associated with TG levels in humans. Among these, Clostridiales and Ruminococcaceae bacteria were positively related to HDL-c levels but not associated with LDL-c or TC levels (38). A recent study reported that Clostridiale was negatively correlated with HDL-c (39). However, our current study did not find a significant correlation between Clostridiale and HDL-c, which may be attributed to the influence of different ethnic groups, study subjects (patients with diabetes, obesity, and MetS) and dietary habits. Moreover, the spectrum of microbiota studied may not be completely extensive, and another microbiota were not specifically measured. Akkermansia is a normal bacterium colonized in the human intestinal tract and is an oval gram-negative anaerobic bacterium. It has been shown that there is a negative correlation between its abundance and overweight, obesity, type II diabetes, and hypertension (40, 41). Some studies have shown that Akkermansia reduced plasma cholesterol indents and the TG and TC levels (42). Akkermansia has also been reported to prevent the development of atherosclerosis (43), consistent with our findings. Thus, the gut microbiota plays a crucial role in the metabolic status of schizophrenia patients. To better understand the microbiome-host correlations, more detailed analysis and more studies are required to determine which bacterial species or strains influence the host.

Some basic and clinical studies have shown that gut microbiota is altered by antipsychotics. Bretler et al. found that obese individuals and those taking olanzapine and risperidone, which eventually leaded to weight gain, have the similar microbiota (44). Olanzapine and risperidone treatment in rodents is associated with reduced gut microbiota diversity and an increased ratio of Firmicutes to Bacteroidetes. These changes parallel those observed in the gut microbiota of obese individuals (44). Concomitant treatment of rats with olanzapine intraperitoneal injections and oral administration of neomycin, etronidazole, and polymyxin (known as “antibiotic cocktail” not only resulted in the reversal of the previously mentioned increased ratio of Firmicutes and Bacteroidetes, but also abated weight gain (44, 45). Another research displayed the agonist of histamine-1/3-receptor (H1R/H3R) could mitigate the weight gain induced by olanzapine in mice (44). The diversity index is higher in first-episode drug-naïve schizophrenia patients compared to those who have suffered from chronic antipsychotic-treated schizophrenia (46).

In this current study, the comprehensive index of α-diversity Shannon index of gut microbiota in the MetS group is higher than the non-MetS group. This findings highlighted different outcomes as compared to the widely accepted notion that a decrease in gut microbiota diversity is linked to diseases (47).The results were similar to that of Vandeputte et al.’s research, which employed 16S rRNA gene sequencing on healthy women (48). Potential reasons for the discrepancy in results could be due to the fact that the subjects involved in the research were diagnosed with schizophrenia, whose gut microbiota composition varies when compared to that of healthy individuals (49). Moreover, gut microbial structure is further modified after antipsychotic treatment (50, 51), such as after being prescribed risperidone for a total of 24 weeks, the genera differed from baseline levels in patients with first-episode schizophrenia (51). Nevertheless, the pathogenic mechanism of these modifications in gut microbiota resulting from antipsychotic drugs uncovers some uncertainties. Alteration in the gut microbiota might increase the susceptibility of hosts to metabolic disorders. Due to the higher risk of metabolic disorders in schizophrenic patients compared to the general population, changes in the gut microbiota exacerbate this metabolic disorder predisposition.

In addition, PICRUSt2 analysis was performed to reveal differences in functional profiles of fecal communities between groups with and without MetS. KEGG analysis showed significant enrichment in metabolic pathways, especially in arginine and proline metabolism. Arginine usually exists in many foods and is involved in various metabolic pathways. It is a substrate for NOS enzymes that generate nitric oxide, a key molecule involved in normal endothelial function and insulin sensitivity (52), exhibiting hypolipidemic activity, particularly in subjects at risk of developing type 2 diabetes (53), improving endothelial (53) and β-cell function (54), and oxidative stress (55). A recent study showed that L-arginine could delay the development of T2DM for a long time (56). Similarly, proline is a unique amino acid whose unique structure distinguishes it from other amino acids in chemical stability and biochemical reactions (57). Proline plays a key regulator role in multiple biochemical and physiological processes, such as synthesizing arginine, polyamines, and collagen and activating mTOR cell signaling. Recent studies found proline could affect food intake and fat accumulation, associated with BP and a possible anti-inflammatory effect (58). These observations are consistent with the consensus that MetS is highly correlated with metabolic disorders and energy surplus. Taken together, the above studies corroborate the role of arginine and proline in metabolic disorders from different perspectives. Herein, we found differences in the metabolism of arginine and proline between the two groups during KEGG analysis based on taxonomic gut microbiota, which supports to some extent the role of gut microbiome in metabolic abnormality. To the best of our knowledge, no previous study has investigated gut microbiome characteristics among schizophrenia patients with MetS. KEGG functional analysis showed that the arginine and proline metabolism significantly reduced gut microbiota among patients with Mets. Furthermore, it indicated that changes in gut microbiota function via the metabolic signaling pathways might contribute to the pathogenesis of metabolic disorders.

The current study has some unique strengths. To the best of our knowledge, this is the first study to investigate the association of gut microbiome with MetS among schizophrenia patients in mainland China. In addition, we comprehensively explored the heterogeneous characteristics of the intestinal microbiome among schizophrenia participants with MetS. Finally, we illustrated the association of the gut microbiota with MetS among schizophrenia individuals. Our findings provide the foothold for further studies on early diagnosis and preventive targets or biomarkers of MetS of schizophrenia patients. Nonetheless, several limitations of our study should be noted. First, it must be mentioned that given that all study participants were Chinese, the results and conclusions should be interpreted cautiously and cannot be extrapolated to other ethnic groups. Accordingly, our findings should be validated in different regions and ethnic groups. Second, this study was a cross-sectional design, and dynamic changes in the intestinal microbiota could not be observed. Hence, additional prospective validation is required to explore further the relationship between gut microbiota and MetS among individuals with schizophrenia. Third, our analysis was not based on the taxonomy of bacterial species. Indeed, different species within a genus may have other effects on metabolism-related parameters. In addition, it should be noted that the sample size of this study is relatively small and the power of the study may be limited. These results should be considered preliminary and need to be replicated in subsequent independent samples. Finally, the microbiota studied in this research mainly came from the lower colon, while the small intestinal microbiota, which plays a significant role in gut microorganism metabolism, was not collected. Therefore, the results are subject to some degree of uncertainty. Given the difficulties in sampling different parts of the intestinal tract in humans, it may be worthwhile to conduct segmented sampling studies in animal models in the future.

In conclusion, we characterized the variations of intestinal microbiome among schizophrenia patients with and without MetS in mainland China. We identified the intestinal microbiome associated with the components of MetS, such as HDL, GLU, and WC and the KEGG pathway associated with the microbiota characteristics of MetS. We also identified specific changes in gut microbiota genera in schizophrenia patients with metabolic syndrome. The identified intestinal microbiome may be harnessed for early diagnosis and preventive targets or biomarkers for schizophrenia patients with MetS.

## Data availability statement

The datasets presented in this study are stored at github. They can be obtained from the following websites: https://github.com/jackzhang83/MetS.

## Ethics statement

The studies involving human participants were reviewed and approved by the research protocol was approved by the Shanghai First Minzheng Mental Health Center (study number: YJZXLL2022022). The patients/participants provided their written informed consent to participate in this study.

## Author contributions

MX: Undertook the data analysis and drafted the manuscript. HG, LY, LW and CZ collected the data, DC engaged in forming the concept and designing the study, supervised by LZ. All authors contributed to the article and approved the submitted version.
